# COVID-19 Misinformation Through the Lens of Young Black and Latinx Michiganders: A Qualitative Study

**DOI:** 10.1007/s40615-025-02646-9

**Published:** 2025-10-31

**Authors:** Susan J. Woolford, Fernanda L. Cross, Ayse G. Buyuktur, Noelle Kellogg, Ziyu Zhao, Rebecca Hunt, Barbara Israel, Sarah Bailey, Erica E. Marsh, Susie Williamson, Kashmira Sawant, Pranati Movva, Jodyn Platt

**Affiliations:** 1 Susan B Meister Child Health Evaluation and Research Center, Department of Pediatrics, University of Michigan, NCRC Building 16, G020, 2800 Plymouth Road, Ann Arbor, MI 48109–2800 USA; 2 School of Social Work, University of Michigan, Ann Arbor, MI USA; 3 Michigan Institute for Clinical and Health Research, University of Michigan, Ann Arbor, MI USA; 4 School of Public Health, University of Michigan, Ann Arbor, MI USA; 5 Michigan Medicine, University of Michigan, Ann Arbor, MI USA; 6 Department of Health Behavior and Health Education, School of Public Health, University of Michigan, Ann Arbor, MI USA; 7 Bridges into the Future, Flint, MI USA; 8 Department of Obstetrics and Gynecology, University of Michigan Medical School, Ann Arbor, MI USA; 9 Corewell Health, Grand Rapids, MI USA; 10 School of Information, University of Michigan, Ann Arbor, MI USA; 11 College of Osteopathic Medicine, Michigan State University, East Lansing, MI USA; 12 Department of Learning Health Sciences, University of Michigan Medical School, Ann Arbor, MI USA

**Keywords:** COVID-19, Misinformation, Social media, Qualitative research, Communities of color, Health disparities

## Abstract

**Background:**

Black and Latinx populations have been disproportionately impacted by COVID-19. While young adults are at lower risk of severe outcomes, they are key to improving the impact of COVID-19 communication in communities of color.

**Objective:**

To understand young Michiganders’ perspectives on misinformation and communication regarding COVID-19.

**Methods:**

In 2021, as part of a larger study, four focus groups were conducted with Black and Latinx residents (18 to 29 years old) in Wayne County (including Detroit), Genesee County (including Flint), Washtenaw County, and Kent County, MI. Using a community-based participatory research approach, we conducted 90-min, virtual focus groups. Key themes were identified using inductive coding.

**Results:**

Six themes emerged from the young adults: (1) reasons for disproportionate impact; (2) social media algorithms propagating misinformation; (3) education helping young adults identify misinformation; (4) “bribery” and incentivizing viewed critically; (5) wide friend networks and diverse messaging providing broader perspectives, but frequent relocation may hamper community connections; and (6) assisting older generations viewed as a responsibility despite not always feeling trusted by their elders.

**Conclusion:**

Participants had a healthy skepticism of COVID-19 information and a sense of responsibility to help their communities. Future work should explore the degree to which young adults may be impacted by misinformation despite their vigilance and how they might contribute to improved health communication.

## Introduction

The COVID-19 pandemic has caused more than 1.23 million deaths in the United States (U.S.), as of August 2025 [1]. Both in cases and deaths, Latinx and Black populations were disproportionately impacted [2, 3]. In addition, Latinx and Black adults over the age of 65 had death rates approximately 2 and 3 times higher than their White peers, respectively [3].

Considering the social and historical contexts, previous studies have identified structural racism as a root cause of higher rates of infection, hospitalization, and mortality among communities of color [2–4]. Factors including residential segregation, wealth inequality, jobs with high risk of exposure, and mass incarceration may impact the ability of individuals of color to avoid risk and access health-promoting resources [2, 3, 5, 6]. This contributes to the disproportionate burden of illness in Black and Latinx communities and to poorer health outcomes as they encounter infectious diseases like COVID-19.

Moreover, research suggests that the health disparities experienced by communities of color are further exacerbated by structural racism and inequities in the U.S. healthcare system, which generate a widespread sense of mistrust of the government and public health authorities [2, 4, 7, 8]. Such mistrust extends into the COVID-19 pandemic and hampers willingness to follow official mitigation and vaccination guidelines, leaving many Black and Latinx individuals to adopt a “wait and see” strategy before accepting COVID-19 vaccines [9].

Additionally, Black and Latinx populations are often the target of misinformation and disinformation, both of which are frequently propagated via digital means, including social media [10, 11]. This likely contributed to concerns within these communities about the best ways to protect themselves in the midst of the pandemic and to vaccine hesitancy.

Although young people 18–30 years of age have lower death rates than older adults, they accounted for more than 20% of COVID-19 cases (the highest percentage for any age group) and experienced significant distress throughout the pandemic [12]. Furthermore, they may be at risk for significant exposure to misinformation due to frequent use of social media and having the highest internet use of any group in the U.S. with almost half stating they are “almost constantly online” [13]. Consequently, special attention to their perspectives regarding the pandemic and communication about COVID-19 is necessary. Little is known about the views of younger Black and Latinx adults regarding effective communication about COVID-19. It is important to identify effective ways to provide Black and Latinx communities with accurate information via channels that they trust. Their perspectives can shed light on the development of culturally appropriate and age-sensitive strategies to encourage vaccine acceptance and to improve health communication in these communities.

Focus groups, as part of the national Community Engagement Alliance (CEAL) project, explored the experiences and perspectives of Black and Latinx young adults during the COVID-19 pandemic, including their insights about sources of COVID-19 communication, factors that mitigate the impact of misinformation, and generational differences regarding how to assess the veracity of health information. In addition, this study addressed actions that may contribute to improved public health communication. The chosen qualitative approach allows our population of focus to express their experiences and perspectives freely and offer a complementary narrative to the extant body of quantitative research regarding the COVID-19 pandemic.

## Methods

This study was conducted as part of the Michigan Community Engagement Alliance Against COVID-19 (MICEAL) initiative, which aims to support solutions to mitigate pandemic-induced healthcare burdens in underserved communities. The MICEAL team adopted a community-based participatory research approach and partnered with a Steering Committee (SC) composed of 15 community organizations and health and human service agencies to develop research questions, focus group protocols, recruitment strategies, data collection, data interpretation, and reports [14].

In 2021, as part of a larger study, four focus groups (*n* = 24 participants) were conducted with 18–29-year-old Black and Latinx residents in four of the counties hardest hit by COVID-19 in Michigan: Genesee, Kent, Washtenaw, and Wayne. This convenience sample of participants was recruited using a snowball approach via email and face-to-face contact. No participants dropped out of the study. The participants were a mixture of those with and without relationships with the study team. Eight participants were from Wayne County, nine were from Washtenaw County, and seven were from Kent County. The majority of participants reported Hispanic or Latino race/ethnicity, and the majority were female. The reasons for conducting the research were shared with the participants, including the aim of the CEAL collaborative.

The focus groups were conducted virtually and each lasted 90 min. Using a pilot-tested guide with prompts, focus groups were conducted by the following female faculty and staff: Woolford, MD, MPH; Cross, PhD, MSW; Buyuktur, PhD, MS, MPH; and Platt, PhD, MPH, all with experience in qualitative methods. Focus groups were conducted via Zoom by two facilitators for each group along with two additional study members present to provide technical support as notetakers without anyone else present. Sessions were recorded.

Inductive coding was used to identify key themes that emerged across the focus groups [15]. A team of four coders analyzed three transcripts, first through pairwise coding and later through a series of full-team discussions. A thematic codebook was developed from weekly full-team discussions throughout the analysis process. Codes were developed, consolidated, discussed, and refined as a team. These weekly team discussions also helped resolve any discrepancies and ensure rigor of qualitative analysis. Emerging themes were presented at monthly SC meetings, and committee members were engaged in data interpretation. Thematic saturation was achieved. One additional transcript was analyzed by the team for the inter-rater reliability (IRR) calculation. Data analysis and IRR calculation (0.7) were carried out in Dedoose. The analysis used a grounded theory approach, and COREQ standards guided the reporting of study methods [16, 17].

All research procedures, including recruitment and consent, were approved by the University of Michigan’s Institutional Review Board (HUM#00190353).

## Results

Six themes emerged from the focus groups as Black and Latinx young adults expressed their unique perspectives regarding the disproportionate burden experienced by their communities and public health communication during the pandemic.

### Reasons for Disproportionate Impact

Participants considered limited information, misinformation, systemic racism, and historical mistrust in institutions as primary causes of the disproportionate impact of COVID-19 in communities of color. Many believed that systemic racism resulted in residential segregation and undocumented immigration, which produced considerable challenges for communities of color to access high-quality healthcare and accurate information to avoid COVID-19 infection given the intimidating cost of medical services and inadequate understanding of COVID-19. One participant stated:

It’s common when an individual gets sick from any one of these communities, they just had to find some home remedies and things to resolve the issue on their own…You don’t realize how big the problem is until you’re scared of it. But it’s like. I don’t want to say too late, but in a sense, yeah. Because at that point, you have to get hospitalized.

Participants noted that discrimination and stereotypes penetrating the medical system further reduced support that individuals of color could receive and thus exacerbated inequities. Participants observed that some medical staff held stereotypes about individuals of color without recognizing the structural barriers they were experiencing:

Medical racism. In medicine where people are saying, there’s this assumption that a lot of Black, Latino, indigenous communities, the reasons why they have health problems… there’s stereotypes and biases around why with heart problems, being obese and having non-healthy lifestyles…There’s a lot of, “You need to do this.” That isn’t paying attention to what’s hard for someone to engage in healthy behaviors if they’re taking care of children, if there’s multiple households in one house, or if there’s so much stress.

Specifically for undocumented Latinx immigrants, participants believed that they possessed a valid fear of engaging with authorities or public health services that could potentially require presentation of legal documentation. This structural barrier inevitably blocked undocumented immigrants’ access to healthcare and COVID-19 vaccination. This theme emerged mainly among Latinx participants. In addition, the presence of a language barrier functioned as another obstacle for Latinx communities to receive correct information regarding COVID-19 public health guidelines. One participant who worked in language services stated that:

A lot of healthcare providers don’t have materials translated in Spanish or other languages. They don’t have interpreters, they don’t have people who can give these people access to the information they need… so many of the announcements that were going on forms and ways to stay safe were not in languages other than English. So that was one thing I thought was really affecting the Hispanic community particularly.

Participants also believed that the historical mistreatment of communities of color by the U.S. government resulted in long-lasting mistrust of the medical system and public health guidelines, leading to community members limiting the information they accepted from these sources. One participant said:

From what I heard about people in my community, especially my mom, they were worried for the same reasons with the way that minorities have been treated in the past. They don’t want it to be them in this instance.

### Social Media Algorithms Propagating Misinformation

Young adults are well aware of social media algorithms and their potential harm by spreading and reinforcing misinformation. Participants acknowledged the openness and diversity of social media platforms like Facebook and Instagram, as information posted by any individual may be accessed by any user at any time without any inspection. Yet, they noted that this facilitates the dissemination of misinformation. In private or semi-private social spaces like WhatsApp, they shared it is particularly difficult to help identify misinformation because user-posted content is less accessible. Moreover, participants were very critical of social media algorithms when talking about COVID-19-related misinformation. They believed that algorithms, which kept promoting similar content to social media users without inspection, would keep people in a misinformation bubble. One participant stated:

Once you’re in one rabbit hole of misinformation, it’s hard to see anything outside of that.

According to the young adults, widespread misinformation on social media and algorithms could play a significant role in shaping people’s mistrust in COVID-19 vaccines and pose serious challenges to public health efforts. One participant said:

People are getting misinformation. I feel like a lot of it comes from our social media, especially with Facebook. A lot of it is also to blame from the algorithms put in place, where people are so used to seeing your spectacle [around the] vaccine, keep on getting information given to you. That’s other people that also are unsure about the vaccine and just keep on going and going until eventually you form the opinion that you’re just not going to get the vaccine because everyone’s saying this, and that.

One participant also mentioned that their peers have lost patience and interest in close reading.

The people in my age group, who have a four year degree, were getting their information from news headlines, not the news stories. They would just actually read the news headlines and that’s what they would reshare.

### Education Helping Young Adults Identify Misinformation

All of the young adults who participated in the focus groups were enrolled in formal education (including high school, college, and graduate school). Participants believed that education was essential for identifying misinformation. For example, higher education allowed young adults to acquire the ability to understand scientific studies, so that they could access and understand the actual medical articles regarding COVID-19 and related health measures instead of trusting messaging from public health authorities. A participant stated:

I actually would just read the actual studies that people were citing because I am lucky enough to understand how people do research. That was the only thing I trusted basically since [the president at the time] took us off from the World Health Organization, which I trusted more than the CDC.

In addition, social science–related coursework in higher education was also viewed as an important resource where young adults were able to learn how misinformation was generated and disseminated through their social networks. Taught in culturally and age-appropriate language, the coursework especially benefited first generation college students from low-income backgrounds and students of color by introducing the systematic way of thinking to debunk false news. A participant who was also a college instructor explained:

I teach a lot of first gen low-income students, students of color, predominantly Latino. I was teaching a social problems class and sociology. I noticed that a lot of them were really receptive to learning about the process, learning about how to debunk false news …They are receptive to these messages and discussions about knowing how their social networks inform them about anything.

### “Bribery” and Incentivizing Viewed Critically

Some young adults reacted strongly to incentives and promotions that were used to encourage vaccine uptake. All focus group participants acknowledged the severity of the COVID-19 pandemic and perceived it as a public health emergency. As a result, they strongly disapproved of incentivizing strategies used by public health authorities to encourage vaccine uptake, including pecuniary compensation, lottery entry, celebrity effect, and what they viewed as potential bribery from health professionals.

Participants viewed pecuniary compensation and lottery entry as means of manipulating emotions that further disadvantaged the communities of color because they undermined the serious nature of the pandemic, promoted public health measures as a game, and failed to provide under-resourced communities with appropriate health education. One representative comment from a participant stated:

It should be more serious and it shouldn’t be like [a] promotion. The state of Michigan has where if you get vaccinated today, you can enter to win millions… If it’s a serious pandemic and it’s a serious crisis, can we take this seriously and not do these weird spin offs and business moves, and that’s where I feel like the disadvantage is with my community. We don’t know whether or not it’s a joke. It’s like they’re tap dancing with us.

Another participant also emphasized the significance of education on COVID-19 vaccines:

I would say just do better education because they’re making it more of like a game… Why don’t you just say, get the vaccine and give actual facts of why you should get it, instead of trying to play on people’s emotions.

In addition, participants criticized public health authorities’ use of celebrities as an easy way to increase vaccine uptake among younger populations. Again, they believed that this strategy transformed a serious public health effort during the pandemic into a fashion trend relying upon the relationship between famous figures and their young followers:

It just didn’t sit right when they would make it like a trend… you know the big creators make it a trend and people follow after that. So then it became a bandwagon; Kendall Kardashian does it, then you know the next person’s doing it, and then the next person, and then everybody in the community wants to do it. People that push out COVID, they knew that they had to get the biggest powers and pay them this X amount of money because they knew that other people will follow after that.

Another incentivizing strategy focus group participants criticized was perceived payment to experts and other public figures to give a specific message. For example, they viewed doctors who appeared in news channels as actors instead of reliable sources of information. One participant stated that:

The one who’s like they go on CNN for what should we do regarding the pandemic, I don’t look to him as a real source of information. I feel like he’s not necessarily an actor, but he’s on the global stage. So it’s like how can you value somebody’s opinion who’s getting paid to say something.

Participants also presented skepticism of doctors and nurses who actually worked in hospitals. The rumors of possible bribery made them suspicious of medical staff’s motives for vaccine recommendation:

And some of the crazy things I’ve heard is that certain hospitals and nurses, and doctors are getting paid to make sure people take the vaccine and stuff like that. So for them to tell me like, “Hey, you need to take this vaccine,” is like, I believe you. You might be coming from a good place, but I also in the back of my mind, I’m like you’re probably getting paid to say this. Like you have to probably meet a quota of how many people you get to get this vaccine.

### Wide Friend Networks and Diverse Messaging Provide Broader Perspectives but Frequent Relocation May Hamper Community Connections

Participants in high school and higher education indicated that they have friends in different parts of the state and all over the world. This wide friend network reduced the impacts of residential segregation and allowed them to obtain information from a variety of resources.

I live in the Flint area, so my friends that go to my school don’t plan on taking it [COVID-19 vaccine]. But I have a lot of friends that live in Canton and the rich parts of Detroit, like the suburbs of Detroit. All the more high-end communities. A lot of them like, ‘Hey, just got my second shot.’ It’s something that they’re proud of. So it just really depends what environment I’m with.

Furthermore, younger adults who attended college were able to leave their home communities and meet people from different social, economic, and cultural backgrounds. Through these diverse social connections, younger adults observed a variety of mitigation practices and attitudes toward COVID-19 vaccines all over the country and the world. Interestingly, these observations made them reflect on the COVID-19-related practices in their home communities. One participant said:

I know there are people dying from COVID, so it’s a serious matter. I have a buddy in Australia and they were affected by COVID. They were on secure lockdown, like you could only go out the house for this amount of hours, but they got over COVID way quicker than us as well too.

Participants generally noticed that students in their universities were more likely to receive vaccines to maintain normal social interactions (e.g., in-person classes and parties), whereas people from their home communities were more hesitant about vaccination. One participant expressed:

I’m pretty diverse. I’m in college. So I have half of people they’re like, no, no way [regarding vaccination], and then half people like yes. Like a lot of my educated friends I go to college with, they all got vaccinated so they could party… but typically not in my community in Flint. I go to [University], so down there, I got some friends that are pretty much on it.

Nevertheless, some participants were concerned about the mobility that came with college life. They viewed this flexibility and mobility as an obstacle for them to build trust in the local sources. One participant stated that:

Within our age range of population, if there is more moving around and relocating for different things, then it’s harder to build trust in the local sources. If you are only in a state for four to five years, and then you head out to a different one.

### Assisting Older Generations Viewed as a Responsibility Despite not Always Feeling Trusted by Their Elders

Focus group participants described older generations in their communities as more likely to be deceived by misinformation. For example, they observed that older generations, who did not have access to formal or higher education, welcomed short, quick messages on Facebook instead of actual scientific studies. Some participants found it challenging to combat misinformation circulating in families because of their parents’ lack of trust in young people’s judgment. Instead, the parental generation tended to invest more trust in doctors with plausibly proven status, even on social media. One participant in graduate school described back-and-forth interactions between herself and her mother:

What they like to hear was if there was a doctor on Facebook that was doing a Facebook live session and was like, “Do this, do that. This is going to help you.”… I would say, “Well, the studies haven’t said this, I understand the studies, I know the process. I can tell you everything that you need to know.” And she’d be like, “I don’t believe you… What they want to hear about is the short, quick messages or the live sessions on Facebook, specifically Facebook that they hear, or the videos that go viral.”

Religion was another factor that participants thought had more impact on older generations than on their own. A strong religious leader may be a reliable source of information for communities of color and, therefore, was able to heavily impact individuals’ attitudes toward vaccination and mitigation strategies. One participant gave an example in Hispanic communities:

I know some pastors were like, ‘Well, it’s going to be okay because God is in control.’ And they kept visiting people and praying for people. And then he got COVID and passed away and it was really sad. A lot of people were getting information from him.

However, it was also pointed out by participants that the older generations were gradually improving their ability to identify misinformation with the help of young people. One participant stated that:

I think the generation of my parents, they’re so accustomed to whatever is put out there is what they’re going to believe, they’re not really going to do much to look more into it. I think now with the pandemic, they’re doing a better job of pointing out, ‘Oh, there is misinformation, especially in the news.’ So they’re starting to slowly utilize the resources they have like iPhones, look up things and just learn for themselves.

Another participant also said,

My family usually will come to me and ask me if they have any questions about things like remedies. And then if it’s something that I haven’t heard about yet, I’ll look it up, and then get back to them on that.

In addition to helping older generations with navigating misinformation, participants felt a collective responsibility to protect vulnerable populations in their communities, particularly the elderly and those with underlying health conditions. They viewed compliance with public health guidelines, including vaccination and mitigation strategies, as crucial contributions to public health achievement and individuals’ well-being in the communities. A participant who reflected the views of many stated:

I’m fully vaccinated and I think that it’s important that everyone gets vaccinated because, just like wearing the mask, it’s a community thing. You don’t only do it for yourself, you do it for others. I know I’m young and I don’t really have any underlying diseases… but I got vaccinated mostly because I was coming home this summer. I was going to be around my parents and my grandparents.

## Discussion

Our findings indicate that young adults are significantly impacted by communication and misinformation related to the COVID-19 pandemic. Participants identified multiple ways in which misinformation rooted and spread, including social media algorithms and limited information coupled with institutional distrust. They also discussed mechanisms by which misinformation could be mitigated, including higher education, wide friend networks, and diverse messaging while sharing a sense of community responsibility and a desire to help lessen the impact of misinformation on their communities through civic engagement.

Participants in our focus groups indicated that education has the potential to mitigate the impact of misinformation and fake news. A few small studies of educational interventions to improve news literacy have supported this idea, showing that age-appropriate educational approaches can positively impact youth’s ability to identify misinformation [18–20]. Our findings emphasize the importance of ensuring that education about identifying misinformation is culturally appropriate, particularly for young people of color and those from low socioeconomic backgrounds.

Our findings also indicated that youth expressed having access to a wider breadth of information through diverse friend networks and relocation. Participants specifically expressed that social influences from others outside of their primary community networks exposed them to alternative social norms and beliefs about COVID-19, including COVID-19 vaccination. Other work has demonstrated that social homogeneity is a significant contributing factor to the spread of misinformation online [21, 22]. This is particularly important to consider within the context of social media algorithms, which perpetuate messaging to users in alignment with already selected content [22]. This can create what one participant described as a “rabbit-hole of misinformation,” in which interacting with misinformation online leads to increased exposure to additional information through an automated algorithm. In recognition of the looming public health threat of misinformation and fake news, these findings indicate the need for tailored, appropriate education regarding identifying misinformation, as well as a need for social media developers and administrators to increase their efforts to reduce the algorithmic spread of misinformation and unmoderated content online.

Participants in this study also discussed their thoughts and feelings regarding incentive programs for COVID-19 vaccination uptake. Current evidence regarding incentives for COVID-19 vaccination uptake is conflicted. Some studies suggest that incentivization is effective when certain conditions are met, while others suggest that incentivization is not a suitable strategy for improved vaccine uptake at all [23–26]. Other work examining perceptions of vaccination incentivization among youth has shown that many have concerns about the use of incentives for vaccination uptake [25, 27]. Our results support this, suggesting that youth felt wary of incentives, particularly those that seemed focused on specific communities or racial groups. Participants described these campaigns that use incentives as manipulative and coercive.

Systemic racism and discrimination was identified as a primary driver of misinformation and inequitable vaccination uptake during the COVID-19 pandemic. This is supported by a body of evidence that recognizes the disproportionate impacts of COVID-19 among communities of color as symptomatic of broader systemic inequities [28–30]. Specifically, undocumented status among Latinx immigrants has been identified in other work as a factor that has created significant barriers to accessing COVID-19 related services, including testing and vaccination [31, 32].

## Implications

Our findings reinforce the importance of providing young people with access to trusted sources of information. For the participants in this study, this typically occurred via schools, universities, and other educational activities, as well as in other spaces young people may congregate. Ensuring access to information through channels aside from social media can aid in combating the misinformation that participants reported on these digital media sources. The results of this study also show the willingness and the power of young people to be leaders working to address the pervasive misinformation found on social media and consumed by the broader community.

The COVID-19 pandemic underscores the need for effective moderation of social media platforms. In addition, transparency with regard to the algorithms used by these platforms should be increased. This may allow people to break free from the cycle of misinformation that can be created by social media algorithms reinforcing misinformation with even more misinformation. Current policy limits the accountability of social media regarding the accuracy of information or the harms that come from misinformation. The COVID-19 pandemic and concerns raised by our participants suggest that misinformation should be considered a public health problem and treated as such [33]. The World Health Organization has adopted the term “infodemic” to describe the spread of misinformation across social media during crisis situations such as a pandemic. They recognize the importance of managing the spread of digital information to avoid harmful, inaccurate messaging [34].

Our results reinforce the importance of repairing harms in communities of color that have been caused by centuries of systemic racism. This must be an ongoing effort, and not limited to times of crisis such as the COVID-19 pandemic. Trust must be established and maintained by public health officials as well as medical professionals in non-emergency situations, so that community members know where to turn during crises. Consistent with our findings about youth, we previously found that people of all ages seek information from multiple sources and need to rely on a range of experts and expertise to be confident in public health, medical, and scientific knowledge [35]. Ensuring that those institutions are also working to explicitly support social justice will extend their credibility and accessibility.

## Limitations

These findings should be considered within the context of certain limitations. The focus groups were conducted in the mid-2021; as such, they only represent a small segment of time during the COVID-19 pandemic. During this time period, information was rapidly evolving, vaccines were beginning to be distributed, and participants’ perspectives may have changed over the course of the pandemic. Participants were more informed than they would have been if these focus groups were conducted in mid-2020, but were also much closer to having lived through lockdowns than if they had shared their experiences in 2022 or 2023. Because these were times of such flux, the experiences recounted here cannot provide a full picture of young Black and Latinx adults’ experiences throughout the pandemic in Michigan. The study data are from a convenience sample of young adults enrolled in school/college in Michigan, and member checking/follow-up interviews were not performed. Thus, our qualitative findings may not be transferable to other populations, including young people who are not enrolled in school/college. Lastly, our sample was largely made up of female and Latinx participants, meaning that their voices may be overrepresented here. However, the study findings contribute to the body of research that reflect the voices of Black and Latinx communities and may inform interventions to enhance health communication efforts to address public health issues such as COVID-19.

## Conclusion

This study enhances our understanding of perceptions about misinformation regarding COVID-19, especially within communities of color. It also highlights potential approaches to address the impact of misinformation within these communities. It underscores the value of including diverse perspectives in conversations aimed at improving health communication. Furthermore, young people, who as a generation have extensive experience navigating social media, may be well positioned to help improve future responses to public health emergencies such as the COVID-19 pandemic.
